# Evo-Devo Algorithms: Gene-Regulation for Digital Architecture

**DOI:** 10.3390/biomimetics4030058

**Published:** 2019-08-13

**Authors:** Diego Navarro-Mateu, Ana Cocho-Bermejo

**Affiliations:** School of Architecture, Universitat Internacional de Catalunya, Carrer Immaculada 22, 08017 Barcelona, Spain

**Keywords:** genetic algorithms, evolutionary development, proto-architecture, visual programming

## Abstract

The majority of current visual-algorithmic architecture is constricted to specific parameters that are gradient related, keeping their parts’ relation fixed within the algorithm, far away from a truly parametric modeling with a flexible topology. Recent findings around genetics and certain genes capable of shape conditioning (development) have succeeded in recovering the science of embryology as a valid field that connects and affects the evolutionary ecosystem, showing the existence of universal mechanisms that are present in living species, thus describing powerful strategies for generation and emergence. Therefore, a new dual discipline is justified: Evolutionary developmental biology science. Authors propose the convergence of genetics algorithms and simulated features from evolutionary developmental biology into a single data-flow that will prove itself capable of generating great diversity through a simple and flexible structure of data, commands, and polygonal geometry. For that matter, a case study through visual-algorithmic software deals with the hypothesis that for obtaining a greater emergence and design space, a simpler and more flexible approach might only be required, prioritizing hierarchical levels over complex and detailed operations.

## 1. Introduction

### 1.1. Definition and Evolution of Architectural Design Through Computational Heuristics

The development of algorithmic-aided design (AAD) [1], or visual programming, is one of the elements responsible for the digital momentum within the architectural discipline, where its accessibility is as important as its potential. Easy-to-use tools available to non-mathematical experts are always part of the discussion of the architectural identity, as the designer is granted new abilities while being simultaneously constrained to them.

Evolutionary algorithms (EAs) have had an amazing impact in the design workflow. By the mid-20th century, Fogel’s ’evolutionary programming’, Holland’s ‘genetic algorithm’ and Rechenberg and Schwefel’s ‘evolutionary strategies’ were already developed [2].

Despite their independent research, a series of evolutionary algorithm conferences in the 1990s allowed beneficial interactions to set the digital evolutionary background, especially within the field of architecture and design. Making such complex algorithms available for non-computational experts through tools linked to visual programming representation allows an exponential growth of the design disciplines regarding the complexity they allow to reach and analyze.

Research on the relationship between evolutionary computation and design has also grown over the last decade. Designers, as Herbert stated already in the 1990s, are trying to improve our understanding by simulating, as imitation is possible because distinct physical systems can be organized to exhibit nearly identical behavior.

Artificial and adaptable systems, considered by the authors as basic elements of the built environment, have rules that make them particularly susceptible to simulations via simplified models. Also, it should be understood that natural sciences are concerned about how the things are, but design must be concerned with how things ought to be, devising “Artifacts” to obtain goals [3].

The evaluation of a particular design can be, as stated by Herbert, done through a series of objective criteria:

1. Theory of evaluation: The utility theory and statistical decision theory.
2. Computational methods:
  - Algorithms for choosing optimal alternatives, such as linear programming computations, control theory, dynamic programming, etc.
  - Algorithms and heuristics for choosing satisfactory alternatives.
3. The formal logic of design: Its imperative and declarative logics.

Concluding that the search for alternatives might be adequate through heuristic search, a theory of structure and design organization, and the development of better representations of the design problems.

Algorithmic solvers allow designers to find out solutions to problems with specific questions which cannot be completely defined. Real world is an extremely dynamic environment for the design process and problems definition; it is non-fully observable, with non-deterministic environments, or environments with some unobservable variables.

Complex situations, such as the ones produced by architecture, can be tackled by computation that can compare and search for huge amounts of results, without really understanding the relationship or the impact between different elements.

### 1.2. Evolutionary Developmental Biology Science (Evo-Devo), Evolution, and Nature Mimicking Algorithms

When geometry, physics, or even society establish unquantifiable relations, is difficult to set a mathematical formula that can solve the question. We shall want to consider systems in which the relationships are more complex than the formal organizational hierarchy. The hierarchical structure of the biological systems is a familiar fact, but, as pointed out in the past by some authors [3], we must establish an important difference between the rest of the disciplines and their hierarchy.

“The most significant advantage of using evolutionary search lies in the gain of flexibility and adaptability to the task at hand.”

[4]

On the other hand, multicellular organisms have evolved through the multiplication and specialization of a single system, rather than the merging of previously independent subsystems.

Evolution is like a complex experiment, with fitness as the sole variable and the structures of individual genes as independent variables. Biology mechanisms can also be thought of as nearly decomposable systems: The interactions within units at any level are rapid and intense in comparison to the interaction between units at the same level.

Despite the discovery at the beginning of the 20th century that homeotic genes can be traced, it was not until 1995 -with the Nobel Prize given to Lewis, Nüsslein-Volhard and Wieschaus- that the revealing of developmental processes also affected by evolutionary changes, and also, that these genes regulate development from the molecular level, started their major impact on society [5].

Despite the discovery of homeotic genes can be traced since the beginning of the 20th century, it was not until 1995 -with the Nobel Prize given to Lewis, Nüsslein-Volhard and Wieschaus- that was revealed that developmental processes were also affected by evolutionary changes from the molecular level; and so, started their major impact on society [5].

This led to evolutionary developmental biology science (evo-devo), which better embraces biologic complexity by relating ancestral gene toolkits to their phenotype, thus becoming a great model for dealing with data and geometry [6]. Developmental biology and evolutionary studies, historically seen as distinct but complementary disciplines, officially merged in 1999 when evolutionary developmental biology, evo-devo, was granted its own division in the Society for Integrative and Comparative Biology (SICB) [7].

However, evo-devo has not been implemented yet in the digital simulation of evolutionary algorithms, which lack many of the geometrical properties related to gene regulation (to be listed and explained later on): Body plan, homeobox genes, genetic switches, and allometric grow [8].

Authors propose the convergence of evolutionary algorithms and evo-devo strategies into a single data-flow that will be proven capable of generating great diversity through a simple and flexible structure of data, commands, and polygonal geometry. To maintain accessibility, the strategy can be incorporated into any optimization algorithm (evolutionary or not) to perform design variety and optimization without the need of coding or additional software.

## 2. Algorithms and Computation in Architecture

### 2.1. Evolution, Emergence, Complexity, and Environment Responsiveness in Architecture

The theory of transformational linguistics and the information-processing theory of cognition were born in the same matrix, produced by the development of the modern digital computer. 

MIT Architecture Machine Group was founded in October 1967 by professor Nicholas Negroponte and was operative until 1985. Creating an “architecture machine” that would help architects design buildings was the very first description of their aim.

Already in 1970, in an article published by Scientific American [9], Conway’s game of life was proposing complexity through a series of simple rules for behavioral relationships. Subsequently, this led to the controversial proposal stated by Stephen Wolfram [10]. 

Similarly, Herbert in 1996 dealt with the idea of an “Artifact” as an interface between the inner and outer environment. The author pointed out that the first advantage of distinguishing two environments was that with just minimal assumptions about the inner environment, we would be able to predict behavior from the knowledge of the system goals and its outer environment. He understood then the outer environment as the one that determines the conditions for goal attainment, while the inner environment as the organization of natural phenomena capable of attaining goals in some ranges of environment. 

In this context, the proposals developed at the Architectural Association by John Frazer [11] are taken as special architectural reference. One of the main worries that stands out from Frazer’s evolutionary architecture is the environment analysis, meaning to develop a responsive architecture that is altered by it, aim that led his interest towards cybernetics.

Picking up the witness, Michael Weinstock emphasizes the biological character of the evolutionary process through projects such as parametric and evolutionary development of urban tissues [12], which serve as precursors to the work later exhibited in this article.

Negroponte also coincides with this aspect:

“… responsive means that the environment which has an active role, initiating to a greater or lesser degree changes as a result and function of complex or simple computations.”

[13]

It can be stated that complexity often takes the form of hierarchy, as hierarchic systems have some common properties independent of their content [3]. How complex a structure is depends a lot on the way we describe it. Since much of design, particularly architectural and engineering design, is concerned with objects or arrangements in real Euclidean two-dimensional (2D) or three-dimensional (3D) space, the representation of space and things in space should be a central topic of research. To achieve simplification, a proper representation must be found. And so, constructing a non-trivial theory of complex systems is by way a theory of hierarchy. Also like this, a large proportion of complex systems observed in nature are hierarchic: The natural world is one where complexity evolves from simplicity.

The notion of substituting a process description for a state description of nature played a central role in the development of modern science. The correlation between state/process descriptions are basic for the survival of an adaptive organism, to its capacity of acting to the environment [3].

### 2.2. State of Computational Genetic Algorithms (GAs) Research in Architecture

Although genetic algorithms (GAs) have been well established and adapted, architects continue to seek strategies for integrating the design philosophy into a functional framework for the evolutionary algorithm. These strategies, understood as autonomous processes, explore architecture without prejudices or constrains, free from subjective judgements; searching for new relations in the design space to find unexpected constructions or different workflows.

“It is precisely the notion of loose control that can be postulated as a possible new authorial model, pitted against the totalizing normativity of current computational design methods.”

[14]

Despite all the virtues of GAs, the majority of current visual-algorithmic architecture deals with changing parameters that are related to specific characteristics. For this reason, the algorithms produce gradients inside those specific characteristic-variable changes within a range.

In that sense, neither their position inside of the algorithm nor the relation between the components changes; the topology stays the same, leading to an algorithm that is not really parametric itself but, rather, fixed.

Results then can be endless and very adaptable, but, rarely unexpected.

Resultant geometries tend to share distinguishing features once the definition has been set. In other words, there are not great leaps in terms of shape grammar inside of the same design. This situation has also led to a detriment in the value of parametric design and the quality of its projects [15].

Crosses between evo-devo and evolutionary algorithm in design have been studied by some researchers for the last 10 years. In 2008, Sprecher and Kalnit, arguing about the tremendous implications that evo-devo could have in design processes, presented research in ACADIA, introducing the idea of recombining switches and shape annealing methods [16]. Researchers were focused on creating a computational structure that was based on a series of subsystems acting as switches, arguing that switch-regulated combinations of subsystems were able to produce a wide variety of morphologies. 

From the 2010s, MIT researchers like Neri Oxman or Skylar Tibbits stated that programmable matter was a feasible future if the intricacies of natural systems investigations were focused properly.

Sidefx Houdini, which combines VDM (Visual Dataflow Modeling) in a procedural generation software, was proposed by Jansen and Chen in 2011 as a possible solution for overcoming the main issues (skills and speed) that used to appear when trying to implement evo-devo design methods. Its main limitation, computer grid accessibility, was proposed to be solved by the EDITS (evo-devo in the sky) method proposed by Jansen in 2013 [17]. This method was supposed to address these three limitations by proposing a system based on Dexen, a distributed execution environment proposed by the author in 2011, a workflow system and a set of computer aided design based design tools.

Nimish Biloria and Jia-Rey Chang developed HyperCell at TU Delft within Hyperbody Lab in 2012 [18]. Tackling existing architectural spaces as a way of investigating how to solve future massive human population growth, they proposed evo-devo as the basis for their Hypercell three main basic ideas: Simple-complexity, geometry rules, and switching/triggering. It was aimed to go beyond structural issues, studying adaptability through space organizations using the idea of the evo-devo’s fate maps.

## 3. Research Hypothesis

As John Frazer stated as far back as 1995, we, as architects, are evolving the rules for generating form, rather than the forms themselves.

As previously mentioned, this research deals with the hypothesis that, for obtaining a greater emergence and variety of the design space, a simpler and more flexible approach might be required, prioritizing hierarchical levels over complex and defined operations. 

A specialized algorithm can only handle the task it has been set to, reducing then variation and unpredictability. In addition, introducing changes later on will be more difficult in reconfiguring the statement [19].

It is evident that communication with the esthetical or philosophical aspect of design is still lacking. A deep gap exists between design’s non-linear approach and the strictly-defined structures of GAs. Moreover, the algorithm remains constrained to the associative topology of the model anchored to the initial design of the architect’s proposal [20].

For this reason, new ways to improve the framework are still being investigated. For instance, recent researches added shape recognition—form diversity—for phenotype evaluation in post-optimization articulation in the genetic process [21]. The addition of dynamic modular frameworks has also been proposed, addressing context for the population—environment, goals, user, etc. [22]— as well as the use of Cartesian genetic programming for modifying the topology of the model [20], or the implementation of interactive cluster-oriented frameworks parallel to the genetic process [23]. 

Authors have tackled, within the brief of a previous research [24], embodied color patterns to translate data, and have used Grasshopper’s path grammar to build a model’s structure in prior articles. 

Following latest AAD trends, this article will focus on the Grasshopper plug-in, based on McNeel’s Rhinoceros3D. Nonetheless, the behavior of visual/graphical/diagrammatic programming or “node editing” can be found in other 3D products such as Dinamo, Maya, Blender, or Houdini.

The purpose of this article is to set the data flow and methodology in visual algorithmic software (such as Grasshopper), in order to incorporate simulated features from evo-devo, allowing great geometric and architectonic diversity in a rather simple algorithm—that is, avoiding the use of scripting or added complexity through external pieces of software (add-ons).

## 4. Digital Abstraction of Evo-Devo: Basic Concept Definitions for Implementation

### 4.1. Bio-Logics of Evo-Devo

From a biological perspective, Darwin’s evolutionary proposal has been constantly updated. The “modern synthesis” theory should be highlighted, which integrates Charles Darwin’s theory on natural selection, Gregor Mendel’s theory on genetic inheritance, mutations, and population genetics [25], along with other compendiums and improvements based on latest discoveries [26].

Current GAs are clearly influenced by the evolutionary theory and have incorporated gene strategy as strings that exchange characters—crossover—mutations to improve the search space and elitism selection through generations. However, they rarely introduce development (shape and change) as a process embodied in the evolutionary search.

Evo-devo also emphasizes more the field of mutations, being one of the elements that puts both evolution and development in common [27]. In spite of negative consequences regarding the efficiency of the search, mutations suppose a great help to transcend “local optima” and to enlarge the “design space”. Pieces of software like Octopus or Wallacei (both add-ons for Grasshopper) include more detailed settings about mutation rate and strength.

#### 4.1.1. Homeobox Genes

Findings around genetics include certain genes capable of shape conditioning (development) that have succeeded in recovering the science of embryology as a valid field that connects and affects the evolutionary ecosystem. They show the existence of universal mechanisms that are present in any living specie, thus describing powerful strategies for generation and emergence. They help to justify the tandem: Evolutionary developmental biology science [6].

The importance of evo-devo is notable. Firstly, for continuing the synthesis of the theory of evolution through embryology and integrating molecular genetics. Secondly, for facilitating the understanding of evolution through the drama of form, illustrating changes and formal diversity [6].

Embryology also explains the natural processes which develop a single-cell into complex multi-cellular organisms. Within DNA, 3% is regulatory. In that percentage, two relevant groups of control can be distinguished: “What” and “where”. Respectively, these are the Homeobox genes and the genetic switches. 

Homeoboxes are genes assigned to purposes, to functionality, directly related to the phenotype; while switches create overlapping patterns that turn those genes on and off. Biologically, there can be up to 10 switches regulating a single gene, from which a functional overlap of patterns can be deduced. This is based then on the concept of emergence and complexity: There are not so many complex tools, but different ways of combining them [28].

As Leroi describes, despite the obvious differences between biology and computation language, these genes work in a way that can be clearly understood [27]:

“The segmental calculator is a thing of beauty. It has the economical Boolean logic of a computer program:

IF ultrabithorax is PRESENT

AND all other posterior homeotic proteins are ABSENT

THEN third thoracic segment: HALTERE”

#### 4.1.2. The Body Plan

These regulatory genes are intrinsically related to a second characteristic of the biologic development: the body plan. 

This mechanism structures and identifies the different parts of the body, allowing self-signaling to interpret the cell’s situation. That is, the body plan can be thought of as the geometric abstraction of the phenotype, a map to order and dispatch the successive transformations, the recursive subdivision in which the switches operate.

When talking about evolutionary jumps, huge changes are justified through multitasking [29]. For this situation to occur, there must be repetitive elements that appear, precisely, because of the body plan. Two different organs might serve to the same purpose, allowing one of them to be specified or changed. Or the other way around, the same organ can be used for two things, as redundancy in the system provides new opportunities (Figure 1).

#### 4.1.3. Allometry

The third tool within the development process is the allometry [30]. Defined as the characteristic which refers to changes in relative size of body parts correlated with changes in overall size, allometry allows organisms with the same topology to end up, for example, with complementary nostrils: A horn versus a blowing hole at the top. However, a thorough analysis will reveal that the topology of the shapes is consistent. 

Allometry allows geometric changes within the same topology, causing a new cycle of modifications on the phenotype, also referred to as “an additional fourth spatial dimension”, with strong proportions related to efficiency, surface areas, and growing patterns [31]. 

In architecture, allometry can be used to speak about scale and proportions, to embed non-linear changes that could be established by the body plan. This would allow adaptation to specific contexts without compromising the topology of the project and its relations.

Digitally, the strength of allometric changes can be applied through lists of boosters/enhancers that resemble typical sliders (numeric ranges) and scale commands. Considering the evolutive analogy, the manifestation of allometry is better related to sliders in the GAs (gene pools) than it is currently to genes.

### 4.2. Geometry Interoperability, 3D Meshes, and Phenotypes

After a conscientious look at the mechanics of evo-devo, some of its similarities with the digital field seem obvious and easily reproducible, such as patterns and Boolean logics. 

From all of them, the most fascinating is the construction and development of embryos, the body plan. Architects will find the geometric functioning of the embryo really comfortable since it is based on orientation, subdivision, symmetry, and polarity.

Together with subdivision, tessellation becomes a notable way to understand inner structures or approach cell aggregation. Whether it be faces, cells, pixels, voxels, or maxels [32], a system that can be subdivided to incorporate hierarchical levels is fundamental to properly simulate the biological aspects.

In that sense, the 3D modeling of polygon meshes seems to be a firm candidate capable of dealing with the needs of the proposed abstraction. Not only because the embryo’s diagram looks surprisingly similar, but because of its advantages over other models such as splines. Mesh lightness and flexibility have been well documented and are currently applied to most software for simulation and analysis: Finite elements, raytracing, collisions, structural models, energy, aerodynamics, 3D printing, etc. Even when it comes to complex surfaces, where NURBS (non-uniform rational B-spline) stand out, meshes are still used in organic modeling due to their subdivision capability.

Therefore, polygon meshes have become increasingly popular in recent years and have great impact in computer graphics and geometry processing. Proof of their versatility is their application to different areas such as neuroscience, mechanical engineering, astrophysics, entertainment, cultural heritage, geo-exploration, architecture, and urban modeling [33].

In that regard, polygon meshes can be valued because of two main advantages with several implications:

- Conceptual simplicity (vertex-edge-face).
  ○ Efficiency for complex systems.
  ○ Better performance for repetitive tasks (such as GAs).
- Topologic flexibility (associative relations).
  ○ Constant subdivision (for organic or hierarchic purposes).
  ○ Relaxation and remeshing.
  ○ Low constrains for geometry modification.
  ○ Fractal properties, since change can apply in different levels.

It is also worth mentioning the universal character of the meshes and a remarkable effort to turn it into a digital raw material. So that, the basic principles and theories keep being updated to perform inside new software such as Grasshopper: Half-edge system [34], implicit surfaces [35,36] or marching cubes [37], are proof of this. In that sense, the Plankton library has special relevance for the Grasshopper community [38].

For all these reasons, the prototype of the case study will rely on meshes, combining data patterns with Boolean logics while modifying them in different levels of their mesh topology.

## 5. Case Study 

### 5.1. Objectives and Setting

The main objective of the case study is to test the viability of building an associative model capable of incorporating the characteristics of evo-devo described above to solve the deficiencies exposed. The definition is designed to resemble a simple path of data, logic and consequent as expected in a digital environment.

The prototype (from now on, individual/s) is formed by a body plan consisting of four to six parts, originally a matrix of 2 × 3 cubes. 

At the same time, new genes with switches and boosters are introduced, ensuring that they can be applied in any of its parts with no repetition limit. That is to say, in some individuals, the same gene can manifest in all its parts. These genes are fully interchangeable and configurable to function in any situation posed by a polygonal mesh. 

Phases of the experiment are as follows, adding complexity incrementally:

1. To treat merely the deformations of the parts (allometry), putting major interest in ensuring that the system works and genes can express freely (switches).
2. Include enhancers (boosters) in those genes through lists, and introduce adaptability criteria to analyze and check their correct evolution.
3. Add modifiers to allow shape changing of the parts, i.e., alter the ‘body plan’, combined with previous phases.
4. Introduce new genes as homeobox (through meshes modifiers) that are expressed in different phases, on top of inner and outer references to the GAs process.

The individuals run through a multi-objective genetic algorithm (MOGA) to better foresee the feasible extremes and possibilities of the definition. Multi-objective algorithms evolve the population in multiple directions (fitness). Three properties are chosen to both maximize and minimize: Area, volume, and number of faces. There are then six fitness criteria that should be contradicting enough to search for different solutions all along the Pareto front.

In that regard, the MOGA is set to 20 generations with a population of 200 individuals, using the default settings established by the plug-in Octopus (SPEA-2): Elitism 0.5, mutation probability 0.2, mutation rate 0.9, and crossover rate 0.8.

**Figure 2:**
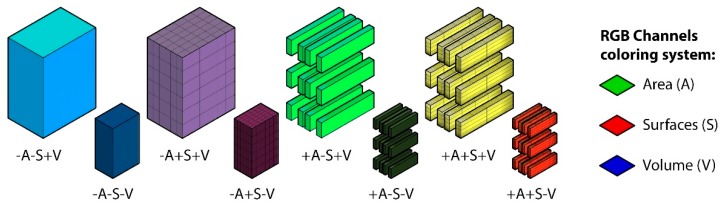
Analysis of individuals based on three fitness criteria both minimized and maximized. Colors applied are based on RGB channels: 30–255, 255 being the maximized value and 30 the minimized. Source: Authors.

Last population after GA implementation is shown as a result of the different phases. Individuals are colored based on their relative properties to better understand their differences and characteristics, better distinguishing the individuals within the population. The coloring system is based on their fitness values, by building an RGB color for each of them: Green (area), blue (volume), and red (faces). Minimized values have darker colors (30, 30, 30), while maximized values tend to be white (255, 255, 255). Pure colors arise with specialized individuals (Figure 2). 

Definitions of genes and which would be the most suitable for architectural construction are left out of the article responsibility, where the most basic ones have been chosen. The case study is meant to remain abstract in order to focus on data flow and component relations. However, it is advisable to consult the pioneer works of Frazer [11], as well late case studies related to the fields of shape grammar [14,38] and space syntax.

To mention a few, most common objectives to be optimized in architecture are: Matter, structure, shadow projection, solar exposure, built area, volume/surface ratio, height, density, connections, or views. Choosing the right genes to influence these properties is key to developing a healthy population and a rich “search space”.

### 5.2. Grasshopper Implementation and Initial Results

The first attempts quickly reveal the first problem: If there is only one flow of data— a typical feature in visual programming—no repetitions of genes can be created (because the component only exists once). Therefore, a path must be created for each part of the individual—up to 6.

To achieve the objective of the experiment, the definition requires that each of the six parts is susceptible to “suffering” each of the genes.

**Scheme 1:**
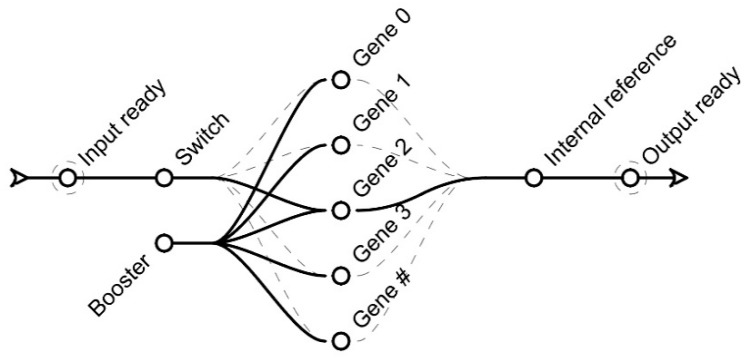
Cluster diagram and path selection through switch. Source: Authors.

For this reason, the inclusion of ‘clusters’ is called for. Clusters are components that incorporate other Grasshopper definitions inside. Each of these clusters incorporates each and every one of the genes. By means of a numerical list indicating which gene has to be expressed, the input geometric information (one of the six parts of the body) is validated only for a single gene, the rest of them resulting in null geometry and producing a null value, non-existent (Scheme 1).

Thus, a model that works exactly as genetic switches is built: Genes are present in all the body, but they only express based on the switch data. There is no restriction on any gene.

Therefore, any hypothetical definition must agglutinate all existing genes into a cluster that will replicate as many times as parts make up the body-plan. There may be fewer parts than the total due to mutations or random elements, but no more, as that would generate an existing but blank information flux. It should be remembered that the modification gene must be universal, i.e., they must be susceptible to be applied anywhere and anytime, otherwise there would be an error in the information flux. As so, the use of meshes in this experiment has one extra justification added.

Regarding other Grasshopper implementations, the definition is based on three simple groups of information. These types will be explained in depth in the following chapters and approached by the different steps of the experiment.

1. The phenotype: Expressed as a mesh, originated in the body plan, that gradually receives changes through genes. It is a list of four to seven meshes to allow independent interaction and modifications.
2. Genes: Composed by modifiers, meaning components that change meshes in different ways: Trimming, scaling (uniform or nonuniform), or remeshing (Mesh+). It is worth remembering that evolutionary algorithms usually consider number sliders as genes. This is not the case, and also the point of this research.
3. Boosters and switches: Both exist as mere lists of data. To relate the genetic algorithm and the Grasshopper definition, these data are originated through a gene pool component (several number sliders grouped under the same constraints).

### 5.3. Steps

#### 5.3.1. To Check Parts Deformation to Ensure that Any Gene Can Be Applied to Any of the Parts

From this point onwards, because of the need to duplicate information, clusters containing gene information are used throughout the algorithm. 

**Figure 3:**
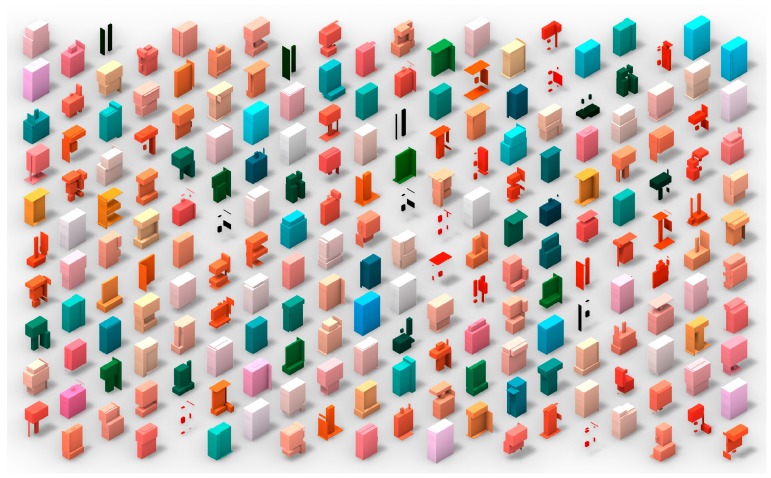
Genes as allometry introduced within a simple body plan. Checking genes repetition and data flow behavior. Source: Authors.

Initial genes only consider scalar changes in different axis, becoming the allometry part of the model. The body plan is still simple and only orthogonal changes are allowed. The Grasshopper’s components for these changes are: Scale, nonuniform scale (Y, Z, and YZ axis), and movement (X axis) (Figure 3).

#### 5.3.2. Addition of Boosters for the Genes Through Lists of Data and Analysis Criteria

The use of numeric lists as parallel information to generate data flow allows to exponentially change possible outcomes of the aforementioned genes. To check the correct behavior of this part of the experiment, an evolutionary algorithm is implemented in the definition (first Galapagos, then Octopus).

As previously mentioned, these lists are based on gene pool components. To improve the search and reduce the design space, these lists include multipliers, i.e., the number of elements within the range borders is reduced: For 0 to 1 there are only five positions, instead of 10 (0.0–0.2–0.4–0.8–1.0).

The individuals produced (phenotypes) soon reach extreme typologies, thanks to the drastic shapes that boosters allow [39]. The diversity found in the Pareto front announces the correct behavior of both body plan and boosters.

Although evolution criteria or settings for the fitness search are not relevant for the final purpose of the experiment, they do confirm basic necessary points for any similar task:

- To observe the extremes and diversity of the population.
- To check that geometry and its values are understood at all times by the GAs.

#### 5.3.3. Addition of Modifiers to Change the Body Plan

**Figure 4:**
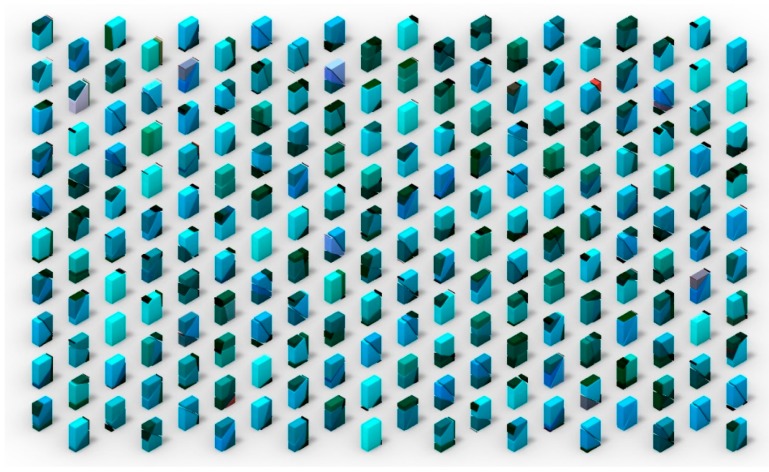
Position and rotation for planes to organize the body plan. Source: Authors.

**Figure 5:**
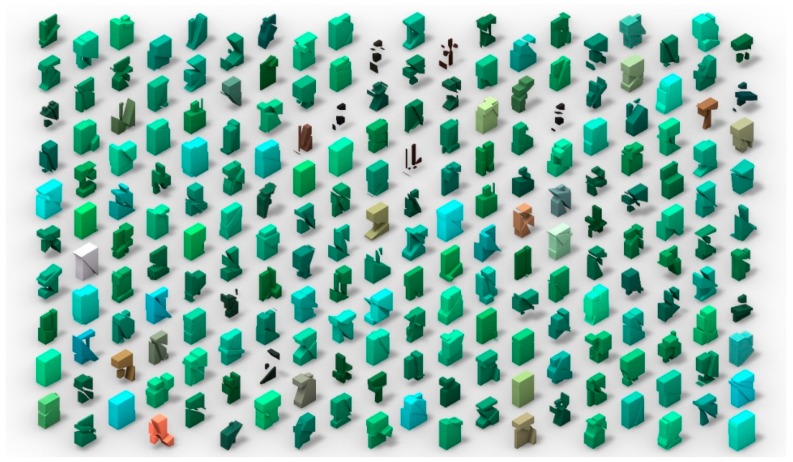
Generation 20 though the test that mixes both allometric genes and non-orthogonal body plan modification. Source: Authors.

To greatly increase the diversity of shapes produced by the definition, the original grid is substituted by a system of moving and rotating planes. These planes divide the basic genotype (an abstract rectangle) in an unknown quantity of parts to be split and extruded as a mesh (ranging from 4 to 7) (Figure 4 and Figure 5). 

To better compare the generations, the original rectangle is kept at 2 × 4 × 6 units. However, introducing genes and clusters in the body plan would also have a positive impact in the richness of the design space. 

#### 5.3.4. Addition of New Genes for Mesh Modification and Their Activation Based on Different Criteria

**Figure 6:**
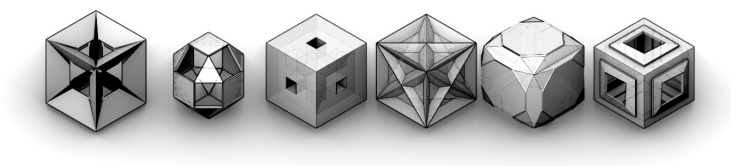
Six different mesh modifiers applied in the last phase to change the topology. Isometric view. Source: Authors.

In this phase, a new toolkit of genes is introduced to change the topology of the parts in the individuals. These mesh modifiers subdivide the mesh and change its geometry by adding, deleting, or connecting their faces. As previously mentioned, there are six homeotic genes that change the phenotype appearance (extracted from Mesh + plug-in): Node, Antisub, Pinch, Hair, Peel, and Polyp. Their only purpose is being able to distinguish the different genes and giving the algorithm more options (Figure 6).

**Figure 7:**
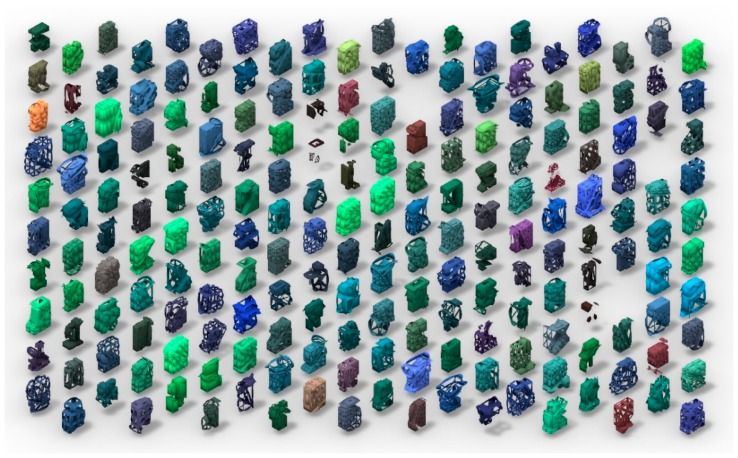
Generation 20 in the last phase of the case study. Source: Authors.

As a second addition to this last chapter, inner and outer references have been introduced to establish different relations to the self-developing system (Figure 7):

- Inner relation has been set within the cluster. When one of the parts has a volume greater than the established number (29 u^3^), the mesh subdivides itself. These should establish a tendency to have biggest parts with more faces.
- Outer relation as a random factor that can influence the individual [11]. To simulate, in a simple way, the environment influence on the phenotype, a probability graph with conditional gates has been set to change the mesh in three ways:
  ○ No changes. 80% probability.
  ○ Catmull-Clark subdivision. 10% probability (subdivision to organic).
  ○ Picture frame. 10% probability (holes in the mesh).

## 6. Results

The use of clusters and universal modifiers ensures fast changes through the parametric model and greatly decreases the size and complexity of the definition. So that, nesting the definition in a hierarchical way has proven useful and a flexible tool.

**Scheme 2:**
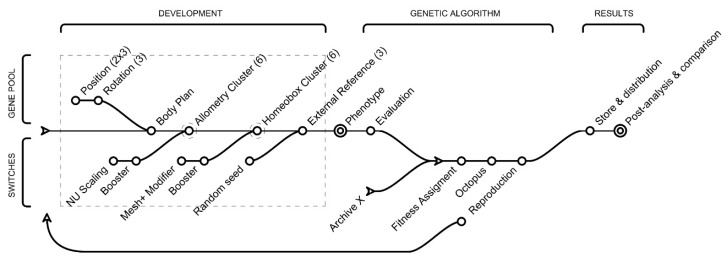
Proposed data flow to parametrize evo-devo within the digital genetic algorithm (GA) framework in visual programming. Source: Authors.

**Figure 8:**
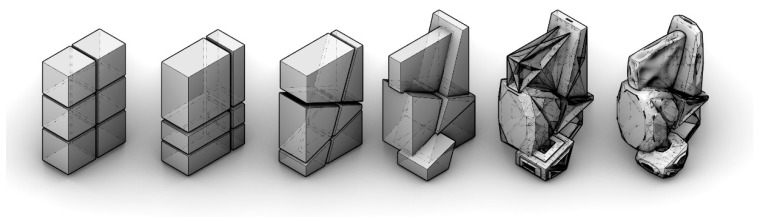
Added complexity into the model (from left to right): Regular subdivision, orthogonal subdivision, non-orthogonal subdivision, allometric changes, application of mesh modifiers, and extra subdivision because environment requirements are met.

Thus, the data flow to simulate evo-devo proposed by the authors after the case study is the following (Scheme 2):

As a result, only two lists of data (gene pools) are needed per gene type: Switch and booster. This procedure allows to stack many levels of genes for different purposes, while the only worry should be that input and output work as a mesh (Figure 8).

It is advised to constrain gradients of booster’s lists to low quantities with significant steps between them, doing the opposite will add unnecessary combinations and slow down the algorithmic search/optimization.

**Figure 9:**
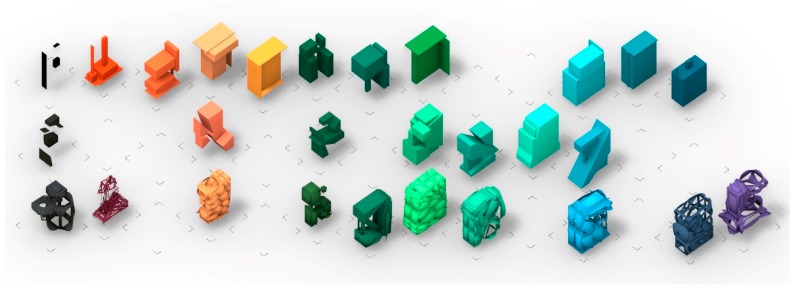
Selection of extreme individuals within the Pareto front. Arrangement based on HUE, each row belongs to a different phase (from top to bottom: First phase, third phase, and last phase). Source: Authors.

The gradient of colors through the phases and additions to the algorithm enhance significative differences. The design space boundaries keep changing, and thus the population tends toward specific hues, some of the colors cannot be reached anymore. Remarkable evaluations from color analysis might be (Figure 9):

- First phase (Section 5.3.1) was able to better optimize geometries related to warm colors, while the last (Section 5.3.4) generation did to cold.
- Phase three (Section 5.3.3), due to the increment of the design space, started to present fewer extreme individuals with a tendency to green (area optimization).
- Last phase (Section 5.3.4) achieved new colors that had not appeared in previous phases, like purple, but lost vivid warm colors.

The evolution in the color scheme makes sense due to the added complexity in the model. Because of this, the algorithm finds it difficult to create individuals with a high amount of faces without increasing areas or volume.

**Figure 10:**
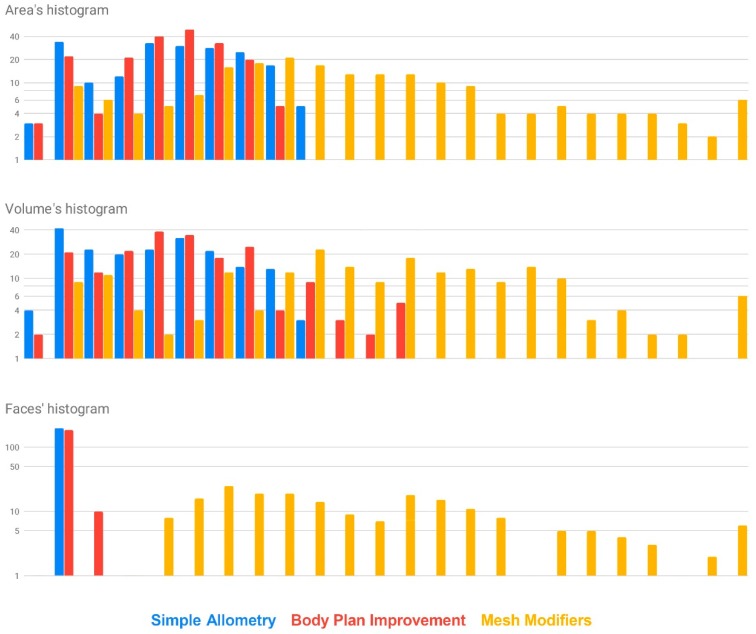
Chart distribution based on data produced by the last generation. Source: Authors.

Three histogram charts (Figure 10) have been arranged to describe the data produced by the last generation of the following combinations. All of them include boosters and MOGA’s optimization. All data in the chart have been normalized to 0–1 in the horizontal axis, while the vertical axis is logarithmic to better describe that very same data.

1. Simple body plan plus allometry (blue).
2. Rotation planes to the body plan plus allometry (red).
3. Mesh modifiers with environment inputs plus allometry and rotation planes (yellow).

Area and volume charts follow the same behavior. It is observable that the successive additions have made it possible to increase the extremes in the maxima while maintaining the minima. That is, design space is increased and repetition is reduced.

Adding complexity to the mesh (red and yellow) was expected to produce both area and volume augment: The first due to the rugosity of the faces, the second because that rugosity increases the bounding box space.

The addition of rotation to the body plan building (red) greatly increased the boundaries, allowing complexity under a less constrained design strategy. The average also decreased, proving again that the population gets diverse.

The third chart (faces) depicts a dramatic change between the mesh modifiers (yellow) and two previous phases. This is expected, as all mesh modifiers increased number of faces and, also, environment setting granted the option to duplicate them. In that sense, it would have been recommendable to introduce mesh modifiers that could reduce mesh face count. Once more, the addition of rotation planes unblocked (red) the capacity of the prototype, that was fixed until then to six-sided boxes.

## 7. Discussion

As architects under the influence of computing, we continue to inquire into its language and the ability of its processes to generate architecture. We continue to explore design and push the limitations of the user interface, supporting that “logic should be the new way” as Leach stated in the 2000s [40].

Although the framework proposed is meant to be as abstract and flexible as possible, architects should always revisit their own understanding for approaching the project, accepting that designing the behavior of the body plan and defining its shape grammar should be part of their design process [41].

The case study has successfully demonstrated the possibility of seamlessly incorporating elements of evo-devo. Approach, strategies, and final design have been considerably influenced by EAs, and the simulation of biological processes allowed to deal with great complexity while keeping advantages and design flexibility.

*“Simply stated, what we are evolving are the rules for generating form, rather than the forms themselves. We are describing processes, not components; ours is the packet-of-seeds as opposed to the bag-of-bricks approach.”*.

[11]

Including a cluster as the body plan was later discarded for a better comparison of the individuals. As in biology, if changes (usually due to mutations) occur in deeper steps of the development, they produce greater modifications. This is highly interesting for discovering new designs, but implementing it in the case study meant too much variance to be properly analyzed later on.

For that matter, architects should always set the boundaries of the body plan within the range of feasibility to reduce unnecessary search through the fitness landscape. Avoiding over information through needless gradients of data will create meaningful genomes with phenotypes that are relevant and can make a difference in the fitness landscape.

### Further Research

Final concerns of the case study were focused towards the implementation in the future of a critical feature from the biologic systems. Phenotypes depend on switches, the body plan, homeotic genes, and boosters [27]; nevertheless, embryological modeling allows to eliminate size association and scaling problems by introducing indirect connections between genotype and phenotype, dramatically reducing computational load [42].

This is crucial in the GA environment, as thousands of individuals have to be expressed as phenotypes for them to be evaluated and selected. Being able to extrapolate from the raw data of the individual (the genome, as in DNA) would have a major impact in the design landscape, as the one calculated in this article, through the addition of evo-devo processes.

The whole process gains special relevance due to the need of reducing the computational load, in the same way that DNA does, trying to avoid the explicit calculation of the phenotype.

The appearance of Building Information Modelling (BIM) might have a great influence in that sense, as the expression of architectural form is generated digitally from information [43]. The study of the physical qualities of a gene has demonstrated, for example, that not every person with the same variation in their genes will physically show on his body a physical abnormality. Those genes out of the normal physical qualities (i.e., extra length on a particular gene) provoke, just in some cases, a physical change in the human body or brain. Because of this, study of the abnormal physical characteristics of the genes in individuals not showing phenotypes variations, confronted by the study of the ones that are indeed showing phenotype variations, has been demonstrated to be a really powerful tool for research on genetic disorders.

The analysis of the physical properties of the genes themselves can be, in a similar way, very useful for analysis purposes when building physical characteristics—phenotype—defined by relevant information systems such as BIM.

On the other hand, keeping a low complexity model of the phenotypes through mesh face memory [44] could help to improve analysis of “architectonic embryos” that would carry on and develop specific properties from basic levels to more detailed ones in the hierarchic structure. Mesh face memory could add inclusive and exclusive properties, or even sub-groups of genetic modifiers within the same parametric model.

Also, as a last matter of further research, the possibility of a machine learning (ML) algorithm implementation has been considered. Once the Pareto front components have been defined, and through the inclusion of certain variables representing the particular requirements for a specific design, a ML algorithm can be taught as the one evaluating and choosing the most adequate element within the Pareto front elements to that very particular design brief. When dealing with explorative generation, this can be a great help in approaching MOGAs and big Pareto fronts.

## Figures and Tables

**Figure 1:**
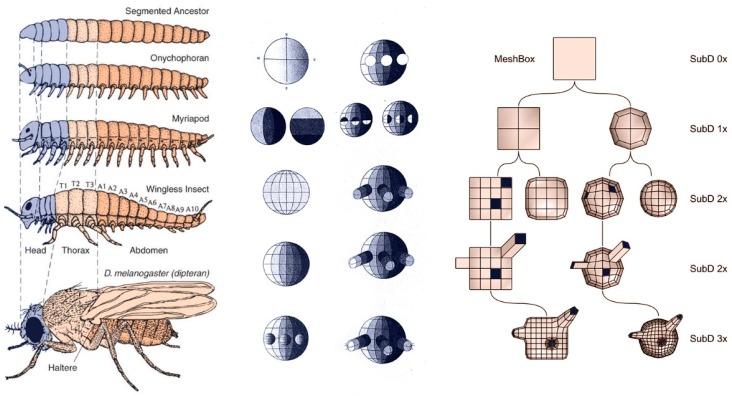
(**Left**) A worm’s body plan. (**Middle**) Body plan comparison between different species and embryo subdivision’s topology (source: Carroll, 2005). (**Right**) 3D mesh subdivision modeling (source: Authors).
